# Protocol for a pilot clinical trial of the senolytic drug combination Dasatinib Plus Quercetin to mitigate age-related health and cognitive decline in mental disorders

**DOI:** 10.12688/f1000research.151963.2

**Published:** 2025-03-05

**Authors:** Abigail Schweiger, Breno Diniz, Ginger Nicol, Julie Schweiger, Andes E. Dasklakis-Perez, Eric J Lenze

**Affiliations:** 1 Social Work, Saint Louis University School of Social Work, St. Louis, Missouri, 63103, USA; 2 Psychiatry, Washington University in St Louis School of Medicine, St. Louis, Missouri, 63108, USA; 3 Psychiatry, University of Connecticut Department of Psychiatry, Farmington, Connecticut, 06030-1419, USA

**Keywords:** Aging, cognitive decline, schizophrenia, depressive disorder, major, cellular senescence, geriatrics

## Abstract

**Background:**

Major depressive disorder (MDD) and schizophrenia are linked to accelerated aging leading to reduced lifespan, health span and cognitive decline. Cellular senescence, in which cells lose proliferative capacity and develop a senescence-associated secretory phenotype (SASP), plays a role in this process. Emerging research suggests that the senolytic regimen of dasatinib+quercetin (D+Q) reduces senescent cells, potentially mitigating age-related health and cognitive decline. This pilot study aims to assess the feasibility and safety of D+Q in older adults with schizophrenia, schizoaffective disorder, and treatment-resistant depression (TRD).

**Methods:**

This single-center study will recruit 30 participants total aged 50 years or older with schizophrenia/schizoaffective disorder or 60 years or older with TRD; the difference in age limits is because individuals with schizophrenia are biologically about 10 years older than general population owing to metabolic burden. Each participant will receive two consecutive days of 100 mg oral dasatinib plus 1250 mg oral quercetin at baseline and weeks one through three, (i.e., two days on, five days off ) along with lifestyle risk management education.

Questionnaires and assessments will measure health and cognitive function as well as psychiatric function at baseline, week 10, and one year. Magnetic Resonance Imaging (MRI) will measure structural and functional brain health at baseline and 10 weeks. Blood sampling for SASP testing will occur at seven time points: baseline, weeks one through four, week 10, and one year.

**Conclusion:**

This pilot aims to evaluate the safety and feasibility of the senolytic regimen and D+Q’s potential to counteract accelerated aging in adults with schizophrenia/schizoaffective disorder and TRD.

**Trial registration:**

Dasatinib Plus Quercetin for Accelerated Aging in Mental Disorders is registered on ClinicalTrials.gov: NCT05838560; posted May 1, 2023.

## Introduction

### Biological degeneration: A convergent mechanism of illness in severe mental disorders (SMDs)

Accelerated biological aging has been proposed as a general pathological mechanism with similarities across mental and physical disorders, necessitating a “convergence” approach to precision medicine that leverages links between physical and mental health. This approach requires new scientific methods that span individual medical conditions with potential overlapping etiologies. Patients with SMDs die younger than non-mentally ill individuals due to earlier onset of cardiometabolic risk conditions like obesity and diabetes, disorders of immune functioning and neurocognitive decline. While mortality in persons with mental disorders is often tied to poverty and associated environmental toxicity that disproportionately effect people with SMDs, the 15-20-year mortality gap is not fully explained by lifestyle and socioeconomic factors alone. Thus, biological aging as a treatment target is particularly relevant in disorders like depression and schizophrenia, which are prevalent and cause significant disability worldwide.

Individuals with schizophrenia experience a greatly reduced lifespan and health span, primarily due to earlier onset of conditions commonly associated with aging, such as diabetes, heart disease, and cognitive decline. ^1^ The causes of cellular aging in schizophrenia are most certainly multifactorial, but in particular, biological risk for the condition itself has been associated with aggressive forms of insulin resistance, for example, which has been proposed as a potential target for new treatments. In Major Depressive Disorder, a subset of individuals – particularly those with TRD, defined as having at least two adequate failed antidepressant trials – exhibit a cellular senescence-associated secretory phenotype (SASP) that can be measured via a simple proteomic test performed on peripheral blood. ^2^ People with depression and a high SASP index value have greater age-related medical comorbidities, ^2^ and their depression is more treatment-resistant. ^3^ These findings coincide with increasing evidence that older adults with TRD are more likely to exhibit signs of accelerated cognitive decline and multiple age-related medical comorbidities. ^4^^–^^6^

### Cellular aging as a treatment target in psychiatric illness

The field of geroscience has proposed that biological aging, underlying age-related mortality and illnesses (such as dementia), is due to a discrete number of aging pathways that are targets for intervention. Of these, cellular senescence has arisen as a key focus for novel interventions (both pharmacological and lifestyle). Cellular senescence is one of the hallmarks of biological aging ^7^ and is characterized by a state of irreversible cell cycle arrest, loss of proliferative capacity, resistance to apoptosis, and a shift in the cellular secretory profile, named as the SASP. ^2^^,^^8^ The SASP is a set of signaling proteins and other biomarkers (e.g., microRNAs, extracellular vesicles) pivotal in governing cellular pathways involved in immune-inflammatory responses, cell cycle control, intercellular communication, and tissue remodeling. ^9^^,^^10^ The SASP is very heterogeneous and probably dependent on the cell type and senescence stimuli. However, prior studies have suggested that few markers are commonly secreted from most senescent cells, such as the interleukin (IL)-6, IL-8, intercellular adhesion molecule (ICAM), FAS-ligand, CXCL-20, MIP-3a, MIF, IGFBPs, among others. Senescent cells are typically cleared from the body by its immune system. The accumulation of senescent cells with advancing age is attributed, in part, to age-related immunological shifts compromising the efficient recognition and clearance of senescent cells. This accrual precipitates detrimental consequences as heightened SASP secretion exacerbates various age-related conditions including cardiac disease, osteoporosis, kidney disease, osteoarthritis, and cancer. ^7^^,^^11^^–^^15^ Moreover, heightened SASP levels have been associated with prevalent aging-related syndromes such as obesity, cardiometabolic syndrome, and frailty, which are also at elevated rates in individuals with mental disorders. ^16^ Notably, the ramifications of cellular senescence and elevated SASP extend to the brain, evidenced by the senescent phenotype exhibited by aged microglia, astrocytes, and neurons in Alzheimer disease affected brains. ^17^^–^^19^

The SASP proteins reflect interrelated biological functions; examining them as a composite index (SASP index) might provide a biomarker for assessing cellular senescence and its response to therapeutics. Our group established a peripheral SASP index comprising 22 proteins. We validated the SASP index and examined it in older adults with depression, ^20^ finding a positive correlation between higher SASP levels and higher comorbid physical health conditions and cognitive impairment in major depression across the lifespan. Although similar studies of SASP have not been conducted in schizophrenia/schizoaffective disorder, existing studies have implicated analogous cellular pathways (e.g., inflammation, oxidative stress) in features of accelerated aging, including advanced brain age.

**Senotherapeutics as treatments for mental disorders – in geroscience and across the age-span.** Senotherapeutic interventions fall into two categories: senomorphics, which reduce the SASP Index value, and senolytics, which selectively hasten the death of already senescent cells. ^21^ A range of senolytic therapies currently exist, spanning behavioral lifestyle interventions (e.g., intermittent fasting) and drugs (e.g., metformin, rapamycin, azithromycin). SASP index levels might both identify individuals most likely to benefit from senolytics and track treatment response (i.e., as senolytics clear out senescent cells from the body, SASP score decreases).

Preclinical studies have found that senolytic therapies can enhance lifespan, health span, and brain function in mice. ^22^ Many of these studies used the combination of dasatinib (a cancer drug) and quercetin (a supplement). ^23^ Both dasatinib and quercetin have shown senolytic effects in different tissues, and it is thought that their combination provides a synergistic senolytic effect. ^24^ These promising results have led to several early-phase clinical trials in various accelerated aging-related conditions, including kidney disease ^25^ and idiopathic pulmonary fibrosis (IPF), ^26^ as well as an ongoing study in Alzheimer disease. ^27^ Importantly, it appears that only brief boluses (e.g., two days of dasatinib+quercetin) are necessary for this senolytic effect; in this dosage, these drugs have been safe and well tolerated in the clinical trials conducted to-date. For example, in an open-label clinical trial including 14 patients with stable IPF, intermittent administration of dasatinib (100 mg) and quercetin (1250 mg) combinitation over 3 weeks was well-tolerated and safe, with most side effects being respiratory and dermatological and classified as mild and moderated. ^28^ Importantly, there was an improvement of pulmonary function and reduction in SASP factors, though they were not statistically significant due to the small sample size of this open-label study.

Senolytics may be particularly relevant to these mental illnesses, given that accelerated brain aging and increased age-related comorbidities are responsible for much of the reduced lifespan and health span. Also, a plethora of work has demonstrated that individuals with serious mental illnesses have significant abnormalities in multiple biological processes related to premature biological aging, ^29^ ^,^ ^30^ including increased cellular senescence burden, ^3^ ^,^ ^31^ ^–^ ^34^ pro-inflammatory changes, ^35^ ^–^ ^37^ and heightened oxidative stress. ^38^ ^,^ ^39^ These pathways are primarily molecular targets for the combination of dasatinib plus quercetin (D+Q), making such combination suitable for testing in individuals with serious mental illness. ^40^ ^,^ ^41^ In addition, both dasatinib and quercetin have demonstrated blood-brain barrier penetrance in rodent models, and there are supportive in human studies; further, preclinical research has demonstrated the efficacy of senolytic agents for neurodegenerative disease. ^27^

In this study, we aim to examine the effects of a combination of D+Q – two drugs with known senolytic properties – on physiological aging parameters in older individuals with schizophrenia/schizoaffective disorder and TRD. To our knowledge, this preliminary study will be the first to test senolytic treatment within the cohort of individuals contending with mental illnesses associated with accelerated aging.

## Aims

**Aim 1: Evaluation of Safety and Feasibility of D+Q in Schizophrenia/Schizoaffective Disorder and TRD.** This aims to assess the safety and feasibility of the combination of D+Q in individuals with schizophrenia/schizoaffective disorder and TRD. Specifically, we will collect data on the rates of adverse events (AEs), serious adverse events (SAEs), treatment discontinuation due to AEs, and treatment completion. Furthermore, overall recruitment numbers will be documented to determine the feasibility of the proposed treatment approach.

**Aim 2: Examination of Changes in SASP with D+Q Treatment**. This aims to evaluate the effect of the combination of D+Q on the SASP in individuals with schizophrenia/schizoaffective disorder and TRD. SASP will be assessed at baseline and weeks one through four to determine acute changes in SASP with each weekly dosing of D+Q, testing the engagement of the treatment target. SASP will also be assessed at week 10 and one year to measure persistence of the treatment effect.

**Aim 3: Investigation of Acute and Long-term Changes in Neuropsychological Functioning, Functional Status, Brain Markers of Aging, and Clinical Symptoms of Schizophrenia/Schizoaffective Disorder and TRD**. This study aims to explore the acute and long-term effects of the combination treatment of D+Q on various aspects of neuropsychological functioning, functional status, brain markers of aging, and clinical symptoms in individuals with schizophrenia/schizoaffective disorder and TRD. Measures of these domains will be assessed at baseline, the end of the acute treatment period (10 weeks for clinical/functional/neuropsychological measures), and the end of one year (all measures) to provide a comprehensive understanding of the treatment outcomes.

The focus for Aims 2 and 3 will be largely descriptive. We do not anticipate inferential testing but plan to examine effect size and 95% confidence interval for effects.

## Protocol

### Trial design

**Figure 1.**
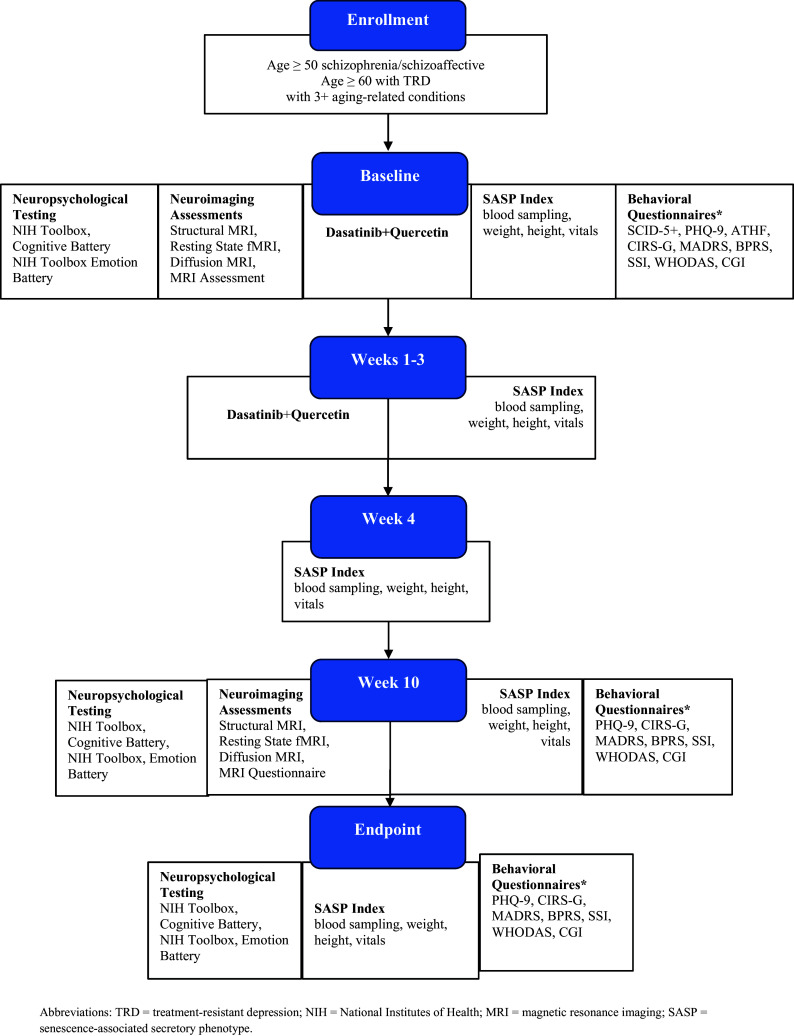
Comprehensive framework.

This single-center, open-label pilot study will be conducted locally through Washington University in St. Louis School of Medicine/Barnes-Jewish Hospital, an academic medical center located in St. Louis, Missouri. The comprehensive trial framework is depicted in Figure 1. The protocol relies on the Standard Protocol items: Recommendations for Interventional Trials (SPIRIT) guidelines.

### Enrollment

Our recruitment strategy will draw from successful approaches utilized in previous clinical trials conducted in our laboratory, leveraging electronic health records (EHR) and the Volunteer for Health platform (VFH). Additional recruitment techniques may include advertisements, media, word-of-mouth/snowballing, and VFH social media. After discussion and pre-screening with the potential participant either by phone or in person, and possibly medical records review or meeting with the patient’s physician, the research team will screen for eligibility per the inclusion/exclusion criteria below. All recruitment materials will be submitted to the IRB for approval.

***Eligibility criteria***

Inclusion criteria will include participants (1) diagnosed with either TRD, defined as major depressive illness with current depressive symptoms despite undergoing at least two adequate trials of antidepressants in this or the previous episode, or schizophrenia/schizoaffective disorder. The rationale for criterion 1 is to ensure that participants have a well-established diagnosis resistant to standard treatments, and the requirement of treatment resistance for depression is due to the findings of elevated SASP and accelerated cognitive decline in older adults with TRD, (2) aged 50 years or older for those with schizophrenia/schizoaffective disorder and 60 years or older for those with TRD. The age criterion is set based on association of reduced health span in these conditions: individuals with schizophrenia have significant age-related pathologies beginning in their 50s or earlier, while individuals with TRD have been shown to have elevated SASP at age 60+, ^42^^,^^43^ (3) presenting with at least three conditions commonly associated with aging which are at least moderate but not end-stage, such as hypertension, diabetes, metabolic syndrome, cardiac disease, non-asthmatic lung disease, adult-onset cancer, arthritis, and other inflammatory diseases typically seen with advancing age. This criterion of multiple aging-related conditions may enrich the sample for elevated SASP, accelerated aging, or benefits from senolytics, and (4) currently receiving an adequate dose of medication tailored for their condition, be it schizophrenia, schizoaffective disorder, or TRD; this ensures that participants are already on a therapeutic regimen, providing a stable baseline for the study.

Exclusion criteria will include (1) contraindications for dasatinib or quercetin, as these are the primary investigational drugs, including concurrent use of other nutraceuticals that might affect outcomes (e.g., urolithin A) (2) active suicidal ideation to a degree where outpatient clinical trial management is deemed unsafe; the safety of participants is paramount, and those with severe suicidal tendencies are not suitable for an outpatient setting, (3) dementia, which is both an end-stage condition and would impede the outcomes and assessments, (4) medications known to be potent CPY3A4 inhibitors or inducers, or those known to induce senescence, such as alkylating agents, anthracyclines, platins, and other chemotherapy drugs, as well as everolimus and topotecan due to their interactions with quercetin. These medications can significantly influence the primary outcomes of the study, and (5) active inflammatory, infectious, or malignant diseases, sensory deficits, recent heart attacks or strokes, severe bleeding disorders, uncontrolled hypertension or diabetes mellitus, active liver diseases, cirrhosis, and current usage of systemic steroids, quinolone antibiotics, hydroxychloroquine, or chloroquine. These conditions will either impair the ability to measure SASP or reflect end-stage conditions unlikely to benefit from senolytics.

#### Informed consent

The Washington University Institutional Review Board (IRB) on April 5, 2023 (IRB ID# 202302203) determined that the study should be designated greater than minimal risk because of the risks associated with the investigational drugs requiring written documentation of consent prior to study participation. The IRB approved a waiver of the requirement to obtain documentation of consent for the phone screen. The principal investigator will conduct and supervise all procedures to recruit participants for the protocol and obtain their verbal informed consent for the telephone prescreening and their written informed consent for all study procedures. Trained study staff will discuss the study, including the risks and benefits of participation, with potential participants and relevant members of their treatment team as needed to provide informed consent for interested individuals. Informed consent will be obtained from all participants before any study procedures are initiated. The consent form, which incorporates The Health Insurance Portability and Accountability Act (HIPAA) authorization, describes the purpose, processes, risks, strategies to minimize them, and possible benefits. Participants will be assured that participation in the study is entirely voluntary and that they are free to withdraw consent at any time and discontinue participation without prejudice to their current or future medical care. The study was approved and received a waiver of documentation of consent from the Washington University Institutional Review Board (IRB) on April 5, 2023 (IRB ID# 202302203).

### Intervention

***Senolytic treatment overview***

The medication regimen for this trial involves the administration of D+Q. Participants will receive two consecutive days of dasatinib 100mg orally plus quercetin 1250mg orally at baseline and weeks one, two, and three (i.e., two days on, five days off ). Thus, each person will receive a total of eight doses of each medication. The D+Q will be self-administered or administered in person. The rationale for this dosing is that brief, pulsed regimens of D+Q have been shown to significantly clear senescent cells and reduce them in the body without causing the toxicity that could be seen with a longer or continuous dosing of dasatinib.

***Lifestyle risk management***

Participants assigned to the D+Q arm will be provided with lifestyle risk management during weeks one through 10 of the trial, in a manner like that conducted in the concurrent study at Washington University. ^44^ To ensure adherence, participants will be brought in weekly to receive their study medication and receive tailored lifestyle education, emphasizing strength, balance, and nutrition. Continuous communication with participants will be maintained to provide support, address concerns, and help navigate obstacles to engaging in the prescribed activities. The rationale for this lifestyle risk management is a necessary element to realize the benefits of D+Q; for example, senolytics may not be expected to show beneficial effects on age-related domains if the participant is highly sedentary and/or eating mainly high-sugar, pro-inflammatory foods. We believe that the lifestyle intervention is facilitatory and reflects the need for a combinatorial approach for targeting aging, rather than a drug-only approach.

We acknowledge the potential need for clinical interventions among some participants, such as optimizing psychiatric medications or intensifying psychiatric treatment. In such cases, recommendations will be made by the study team on a case-by-case basis, prioritizing participant safety. Our specialized knowledge, particularly in psychiatry, will ensure the effective execution of this plan:

1. Participants receiving ongoing care from a provider (PCP, psychiatrist, therapist): We will advise these participants to continue their current care, while being available to address any inquiries regarding their ongoing management.
2. Participants who report to us that they are in crisis, such as severe worsening of symptoms: We will assess the severity of the crisis and implement measures necessary to ensure their safety and proper management.

### Data collection

While participant compliance with data collection at all time points is anticipated, non-completion of certain assessments will not be grounds for exclusion. Furthermore, to promote participant retention and follow-up completion, we will have inpatient as well as telephone contact with participants throughout the acute phase and until the one-year mark.

***Neuroimaging***

Participants will undergo neuroimaging assessments at baseline and 10-week. These assessments involve a brain MRI lasting approximately one hour, administered on a Siemens 3T Prisma Scanner at Washington University. The imaging protocol will include both anatomic-related brain structures (e.g., hippocampal volume) and functional (resting state fMRI) modalities. Structural MRI will assess brain health and aging, while resting state fMRI will assess whether brain connectivity changes are seen with D+Q (providing a CNS measure of target engagement or treatment mechanism). In addition, diffusion MRI will be conducted, comprising two scans per sequence with b values of 1500 and 3000, featuring 2.0mm voxels, TR=3500ms, TE=83ms. Should any clinically meaningful abnormality arise or necessitate further medical attention, workflows have been established for sharing the MRI images and reports with the participant’s healthcare provider.

***SASP index***

Participants will provide blood samples at seven time points: baseline, weeks one through four, week 10, and one year (for those completing D+Q regimen). Blood draws for weeks one through four will be scheduled for subsequent week post-dosing. For instance, if a participant initiates their study medication on Monday, their week one blood draw will occur the following Monday. Each subject will contribute approximately 10 ml of blood, with accompanying measurements of weight, height, and vital signs. These blood samples will undergo analysis for SASP and other yet-to-be-determined blood-based markers. SASP proteins hold promise as diagnostic markers for the accumulation of senescent cells and subsequently age-related pathologies. Reflecting interrelated biological functions, SASP proteins, when evaluated as composite biomarker index (SASP index), present a robust measure for assessing cellular senescence and therapeutic responses. While SASP expression is cell-dependent, a core set of SASP proteins is commonly expressed by senescent cells. Our research team has developed a peripheral SASP index comprised of 22 proteins. This is a target engagement test – changes in SASP reflect target engagement, assuming that they correlate with changes in the clinical and neuroimaging outcome (e.g., the greater the reduction in SASP, the greater improvement in cognitive performance). If we find changes in SASP with dosing of D+Q, this may indicate that SASP levels can be used to track response to treatment.

***Neuropsychological testing***

The neuropsychological evaluation encompasses the NIH Toolbox Cognition Battery, supplemented by paragraph recall, alongside two psychological well-being assessments from the NIH Toolbox Emotion Battery. These assessments are slated for three specific intervals: baseline, week 10, and study endpoint. While the Cognitive Battery is specifically tailored to assess participants’ cognitive functions and memory recall capabilities, the psychological well-being assessments aim to gauge their mental health and overall well-being. The results of these assessments will serve to establish a neuropsychological baseline and subsequently monitor the participants’ cognitive functions and psychological well-being throughout the study. This approach aims to discern the intervention’s impact on the participants’ cognitive abilities and emotional wellness. All research team members undergo comprehensive training to administer neuropsychological testing. This includes practice sessions, protocols for scoring and regular assessments of interrater reliability.

***Behavioral questionnaires and assessments***

**Figure 2.**
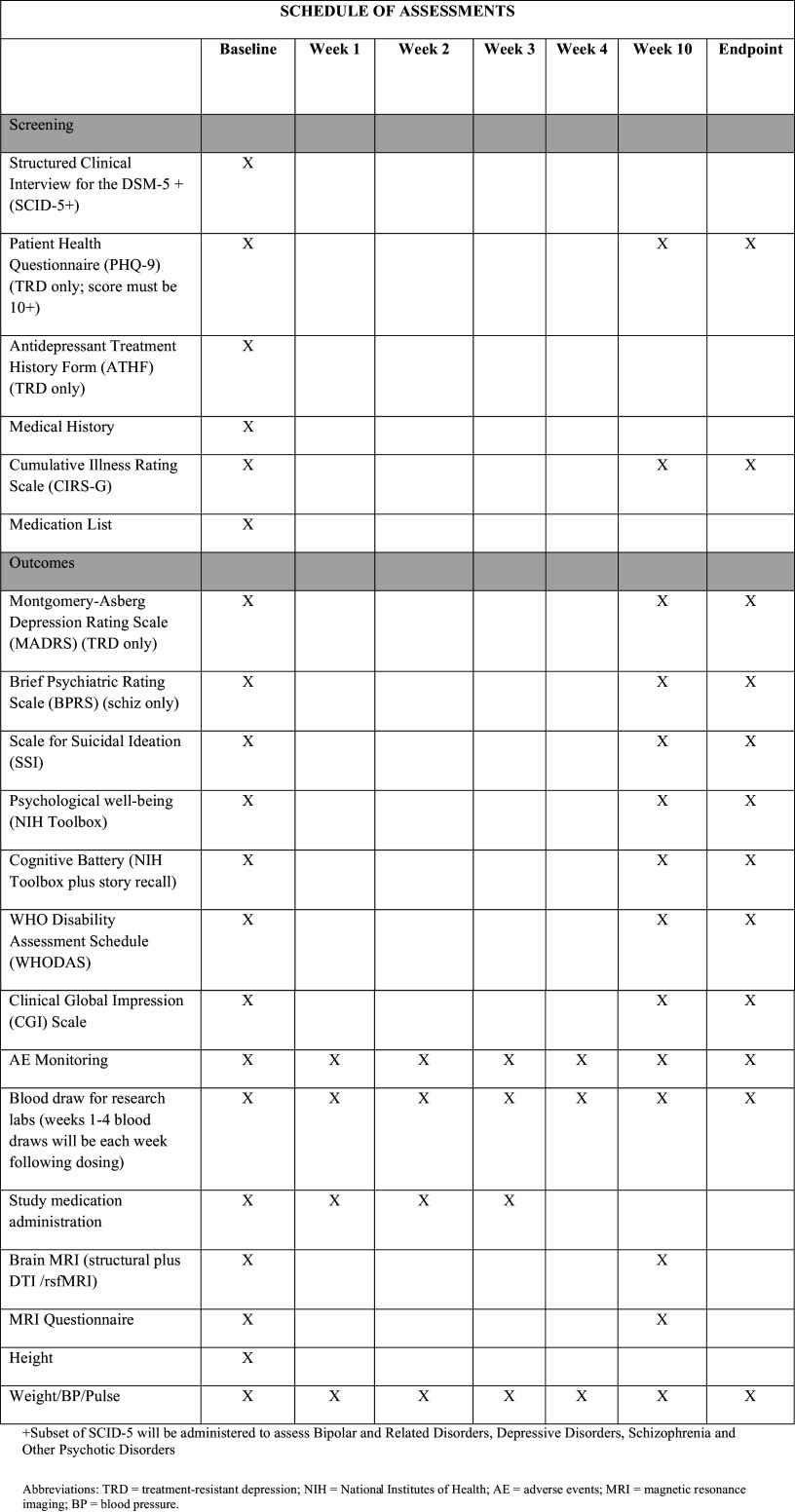
Schedule of assessments.

Throughout the study duration, participants will complete a series of assessments to evaluate their health status and mood, including both self-report and clinician-administered measures. Self-report measures, such as the Patient Health Questionnaire (PHQ-9), NIH Toolbox Psychological Well-Being Scale, and WHO Disability Assessment Schedule (WHODAS), will be completed independently, while all other assessments will be clinician administered. These assessments will be administered at baseline, week 10, and the study’s endpoint. Phlebotomists will conduct blood draws and record vitals, MRI technicians will perform MRI scans and administer the MRI safety screener, and the rater or study coordinator will administer all other clinician rated assessments. To ensure consistency and accuracy in data collection, all research team members will undergo comprehensive training, which includes practice sessions, adherence to standardized protocols, and regular evaluations of inter-rater reliability. Inter-rater reliability will be maintained through double-scoring sessions and periodic reviews of scoring pradtices. The schedule of assessments is shown in Figure 2. The subjective data from the questionnaires, along with the objective findings of the neuroimaging and biomarker analyses, will present a holistic understanding of the intervention’s impact across common mental and physical health domains.

### Study intervention risk profile and mitigation strategies

***Treatment with D+Q***

D+Q in tandem have potential adverse effects. While prolonged daily use of dasatinib carries potential for several serious adverse effects, this study administers only eight doses intermittently, like other studies of these drugs as senolytics. Furthermore, the dose that will be used in this study is considered safe and there are no interactions between quercetin with dasatinib. ^45^^,^^46^ From the three clinical trials that have employed intermittent D+Q dosing, only one instance of a SAE has been documented: edema and pleural effusion. However, the causal relationship between the medication and the SAE remains inconclusive. Non-SAE reported during these trials included respiratory symptoms, skin irritation and ecchymosis, as well as gastrointestinal discomfort. ^25^^,^^26^^,^^47^ These known risks associated with this therapeutic combination are categorized as follows:

• **Likely:** Cough, nausea, and headaches.
• **Less likely (1-10%):** Shortness of breath, runny nose, respiratory infection, fatigue, dizziness, anxiety, sleeplessness, skin irritation, bruising, appetite changes, constipation, diarrhea, heartburn, joint pain, either dry or watery eyes, as well as mouth soreness.
• **Rare (<1%):** Edema and pleural effusion.

To mitigate the associated risks of D+Q, the study adopts an intermittent dosing regimen, administering eight doses over a four-week period. Oversight by expert medical personnel ensures participant safety and appropriate medication management. It is pertinent to note the potential fetal toxicity linked to dasatinib. ^48^ Despite the study’s inclusion criteria stipulating an age threshold of 50+ for the schizophrenia/schizoaffective group and 60+ for the TRD group, all participants will receive comprehensive information regarding the fetal risks associated with dasatinib. Women of child-bearing potential and their male counterparts are strongly advised to employ effective contraception measures, such as condoms, throughout the study and for 90 days following the final dose.

***Dose adjustment***

In the event a participant becomes pregnant or develops intolerable side effects during the treatment phase of the study, we will discontinue the study medication. Participants can continue with the other study assessments that are not contraindicated by pregnancy.

***Lifestyle intervention***

The content of the healthy lifestyle intervention includes weekly health coaching content with information on healthy diet and physical activity adapted from an existing intervention developed for delivery in community clinical settings.

***Peripheral biomarker collection***

While generally safe, peripheral biomarker collection sometimes results in discomfort or minor complications at the venipuncture site. To optimize outcomes, only qualified personnel are authorized to conduct blood draws, adhering rigorously to sterile techniques and institutional policies to ensure participant safety.

***Neuroimaging procedures***

Neuroimaging procedures, particularly MRIs and fMRI scans, carries inherent risks for participants, including potential discomfort and feelings of claustrophobia during the scan. Additionally, certain metal implants or medical devices may impede the imaging process, posing a risk to the participant. To ensure participant safety, individuals with MRI contraindications are excluded from this aspect of the study. Pre-scan screening is conducted to identify any metallic objects within the body, with their presence warranting exclusion from the imaging procedure. Participant comfort is paramount, with provisions made for pauses, rests, or opt-outs should discomfort arise. Earplugs are provided to mitigate scanner noise. In the event of significant abnormalities detected during the scan, participants will be promptly referred to their primary care physician for further evaluation and treatment.

***Risk-benefit assessment***

The study’s significance lies in its potential to inform future research. By investigating potential treatments aimed at counteracting accelerated aging in individuals with mental illness, the study aims to bridge a critical knowledge gap regarding accelerated aging in mental illnesses. Direct clinical benefits to individual participants are not guaranteed. However, participants will undergo comprehensive assessments at the study’s initiation and regular intervals thereafter. These assessments hold the potential to reveal previously unidentified conditions, such as acute medical or psychiatric illnesses. Addressing these conditions could lead to enhanced health management strategies for participants, thereby offering avenues of care that may have otherwise remained unexplored without the study’s intervention. Low risks associated with participation juxtaposed against the potential for both scientific advancement and individual benefits suggest a favorable risk-to-benefit profile.

### Data management and analysis

***The overall aim of this randomized pilot and feasibility study is to determine safety, tolerability, and detection of early treatment effects in***

1. **Safety and feasibility of D+Q:** The primary aim of the study is to evaluate the safety and feasibility of D+Q in two mental illnesses associated with accelerated aging: schizophrenia/schizoaffective disorder and TRD. To achieve this, the study will collect data on rates of AEs, SAEs, treatment discontinuation due to AEs, treatment completion, and overall recruitment numbers.
2. **Changes in SASP with D+Q treatment:** The study seeks to examine changes in the SASP consequent to D+Q administration. SASP dynamics will be examined at multiple time points to discern acute and long-term changes.
3. **Neuropsychological functioning, functional status, and brain markers of aging:** The study will investigate acute and long-term changes in neuropsychological functioning, functional status, brain markers of aging, and clinical symptomatology across illnesses. These parameters will be assessed at baseline, the conclusion of the acute period (10 weeks) for clinical, functional, and neuropsychological measures, and at the end of one year, allowing a preliminary view of the intervention’s impact.

***Analytical approach***

The analysis for Aims 2 and 3 will be primarily descriptive in nature. The study does not anticipate inferential testing. Instead, the focus will be on examining the effect (in terms of changes in measures) and providing a 95% confidence interval for the observed effects.

This approach ensures that the data analysis is aligned with the study’s primary objectives and provides a comprehensive understanding of the impact of D+Q on the mental illnesses of interest. Using multiple time points for assessment ensures that immediate and long-term effects are captured, providing a holistic view of the treatment’s efficacy.

While not appropriate for this feasibility trial due to the small sample size, a future trial would implement a mixed model repeated measures ANOVA with a treatment group by time point design to determine the overall impact of the intervention on the change in the outcomes. The mixed model ideal because it uses all available data and is robust despite missing data.

### Dissemination

The findings from this trial will be disseminated through scientific forums, such as conferences and peer-reviewed journals. Target audience includes primary care physicians and psychiatrists. Furthermore, the main outcomes will be made publicly available through ClinicalTrials.gov, adhering to the International Committee of Medical Journal Editors’ guidelines on authorship for published articles.

### Study status

The study is ongoing, and no data analysis has been performed.

## Discussion

This study will be the first to test a senolytic strategy for mitigating accelerated aging effects in older adults with schizophrenia/schizoaffective disorder and TRD, who are at high risk for reduced lifespan, health span, and accelerated cognitive decline. Owing to its first-in-kind nature, this clinical trial takes an important first step in exploring the effects of senolytic therapy on a known biomarker of cellular senescence and clinical outcomes. However, for the same reasons, the study will be subject to important limitations. First, the study is open-label and has a small sample size; results will be preliminary, and future studies will need a control group and likely more statistical power to measure treatment effects. Moreover, in an open-label study the awareness of both participants and investigators regarding the administered treatment raises the risk of expectancy biases. Also, the participant cohort recruited for this study may not entirely represent the broader population of individuals with the targeted conditions. Another salient limitation is the duration of follow-up. Despite the inclusion of multiple assessment time points, including a one-year follow-up, the long-term effects of D+Q remain largely unexplored; Therefore, we do not know the best duration of study to assess treatment effects.

Looking forward, several avenues for future research emerge. For example, some have argued that clinical benefits of senolytics may be transient at best if biological mechanisms that extend the life and function of remaining healthy cells, like telomerase activity, are not simultaneously addressed, potentially accelerating degenerative processes in the long-term. ^21^^,^^49^ Similarly, when we focus on suppressing biomarkers of senescence, we risk conflating biomarkers with aging when they are simply signs of illness in the face of impaired maintenance. Thus, larger randomized controlled trials that measure cellular mechanisms associated with senescence and with maintenance of cellular functioning together with clinical outcomes, would provide more robust evidence concerning the efficacy of D+Q as a senolytic and/or senomorphic treatment. Extended observational follow-up of clinical trials and observational examination of health systems data tracking relevant outcomes across relevant age and diagnostic cohorts spanning several years will also be needed to elucidate the long-term safety and efficacy of the intervention.

Nutraceuticals-naturally occurring compounds with therapeutic potential – is also another option that has gained growing attention for managing psychiatric conditions associated with accelerated aging. Omega-3 fatty acid, curcumin, and co-rich products are examples of naturally occurring compounds with anti-oxidant and anti-inflammatory effects. While D+Q directly targets senescent cells, the addition of nutraceuticals may provide supplemental benefits regarding inflammation and oxidative stress, potentially amplifying the benefits of D+Q in those with mental illness. Furthermore, future studies should continue to examine the synergistic effects of combined senolytics and nutraceutical therapy to optimize outcomes for individuals with psychiatric disorders and accelerated aging.

Senolytics are not going to cure death but administered with precision (given to the right person for the right problem at the right time), they could extend healthy lifespan to a degree that allows middle-aged and older adults to lead longer and more productive lives. This is of increasing public health importance, as birth rates decline and the proportion of the population that is aging continues to grow. This study lays the groundwork for understanding the effects of D+Q. Exploring D+Q in combination with other pharmacological and non-pharmacological therapies may yield synergistic benefits. Future studies will benefit from the inclusion of a diverse participant pool, considering factors like age, gender, ethnicity, and comorbidities. This inclusivity offers insights into the differential treatment effects across various population subgroups. Such studies would allow enhanced generalizability of results while also appropriately interrogating mechanisms of illness and health across conditions and ages. Beyond clinical and neuropsychological outcomes, future research should incorporate considerations of the treatment’s impact on participants’ overall quality of life and health span.

## Conclusions

This study probes safety, feasibility, and prospective therapeutic effects of D+Q for individuals with schizophrenia/schizoaffective disorder and TRD. By examining treatments to mitigate the relationship between accelerated aging and mental disorders, we aim to provide insights into the reduced health span and lifespan in these disorders. This provides a novel path for reducing the morbidity of common mental disorders.

### Ethical considerations

*All personnel involved in the design and conduct of the research involving human participants will receive the required education on protecting human research participants before the start of the project. This study has been approved by the Human Research Protection Office at Washington University (approval #202302203) on April 5, 2023. All study procedures will comply with the Washington University IRB informed consent standards. Any protocol modifications will be approved by this IRB, and significant modifications will be communicated by updates to the trial registration and/or direct communication with participants. The study has also been registered at* ClinicalTrials.gov *(NCT05838560, posted May 1, 2023). Informed consent will be verified in writing with all participants prior to their enrollment in the study. Through the consent process, those being enrolled will receive education on the voluntary nature of their participation as well. The informed consent form that will be used and the completed SPIRIT checklist are available as Extended data.* The Washington University Institutional Review Board (IRB) determined that the study should be designated greater than minimal risk because of the risks associated with the investigational drugs requiring written documentation of consent prior to study participation. The IRB approved a waiver of the requirement to obtain documentation of consent for the phone screen.

*Data confidentiality*

While rigorous precautions are enforced, the study acknowledges the potential for inadvertent breaches of electronic or paper records breaches. To counteract such vulnerabilities, stringent data management protocols are employed. These protocols include utilization of locked storage facilities, the implementation of restricted data access controls, and the adoption of anonymized participant identification numbers. Such measures are thoughtfully designed to safeguard the integrity and confidentiality of participant data throughout the study.

## Data Availability

No data are associated with this article. Open Science Framework: ‘Protocol for a pilot clinical trial of the senolytic drug combination Dasatinib plus Quercetin to mitigate age-related health and cognitive decline in mental disorders,’ https://doi.org/10.17605/OSF.IO/92GMQ. ^50^ This project contains the following extended data:
- Senolytics Informed Consent.pdf - SPIRIT checklist for ‘Protocol for a pilot clinical trial of the senolytic drug combination Dasatinib plus Quercetin to mitigate age-related health and cognitive decline in mental disorders.pdf’ - Study forms and assessments Data are available under the terms of the Creative Commons Zero “No rights reserved” data waiver (CC0 1.0 Public domain dedication).

## References

[1] Seeman MV : Subjective Overview of Accelerated Aging in Schizophrenia. *Int J Environ Res Public Health.* 2022;20(1). 10.3390/ijerph20010737 36613059 PMC9819113

[2] Seitz-Holland J Mulsant BH Reynolds CF III : Major depression, physical health and molecular senescence markers abnormalities. *Nat Mental Health.* 2023/03/01 2023;1(3):200–209. 10.1038/s44220-023-00033-z 39483500 PMC11527398

[3] Tessema T Diniz BS Vieira EM : Elevated senescence-associated secretory phenotype index in late-life bipolar disorder. *J Affect Disord.* 2024;360:163–168. 10.1016/j.jad.2024.05.071 38795779 PMC11209851

[4] Barzilai N Cuervo AM Austad S : Aging as a Biological Target for Prevention and Therapy. *JAMA.* 2018;320(13):1321–1322. 10.1001/jama.2018.9562 30242337

[5] Diniz BS Lavretsky H Karp JF : Mood Disorders and Dementia: Time for Action. *Am J Geriatr Psychiatry.* 2020;28(5):542–544. 10.1016/j.jagp.2019.11.009 31843380

[6] Reynolds CF III Dew MA Pollock BG : Maintenance treatment of major depression in old age. *N Engl J Med.* 2006;354(11):1130–1138. 354/11/1130 [pii]. 10.1056/NEJMoa052619 16540613

[7] McCulloch K Litherland GJ Rai TS : Cellular senescence in osteoarthritis pathology. *Aging Cell.* 2017;16(2):210–218. 10.1111/acel.12562 28124466 PMC5334539

[8] Diniz BS : The Molecular Intersection Between Senescence and Major Depression in the Elderly. *Am J Geriatr Psychiatry.* 2018;26(11):1097–1105. 10.1016/j.jagp.2018.07.005 30150070

[9] Wang B Han J Elisseeff JH : The senescence-associated secretory phenotype and its physiological and pathological implications. *Nat Rev Mol Cell Biol.* 2024;25:958–978. 10.1038/s41580-024-00727-x 38654098

[10] SenNet C : NIH SenNet Consortium to map senescent cells throughout the human lifespan to understand physiological health. *Nat Aging.* 2022;2(12):1090–1100. 10.1038/s43587-022-00326-5 36936385 PMC10019484

[11] Coppe JP Desprez PY Krtolica A : The senescence-associated secretory phenotype: the dark side of tumor suppression. *Annu Rev Pathol.* 2010;5:99–118. 10.1146/annurev-pathol-121808-102144 20078217 PMC4166495

[12] Farr JN Khosla S .: Cellular senescence in bone. *Bone*. 2019/04/01/2019;121:121–133. 10.1016/j.bone.2019.01.015 30659978 PMC6485943

[13] Mehdizadeh M Aguilar M Thorin E .: The role of cellular senescence in cardiac disease: basic biology and clinical relevance. *Nat Rev Cardiol*. 2022/04/01 2022;19(4):250–264. 10.1038/s41569-021-00624-2 34667279

[14] Wang WJ Cai GY Chen XM : Cellular senescence, senescence-associated secretory phenotype, and chronic kidney disease. *Oncotarget.* 2017;8(38):64520–64533. 10.18632/oncotarget.17327 28969091 PMC5610023

[15] Wang Z Gao J Xu C : Tackling cellular senescence by targeting miRNAs. *Biogerontology.* 2022;23(4):387–400. 10.1007/s10522-022-09972-z 35727469

[16] Schafer MJ Zhang X Kumar A : The senescence-associated secretome as an indicator of age and medical risk. *JCI Insight.* 2020;5(12). 10.1172/jci.insight.133668 32554926 PMC7406245

[17] Flanary BE Sammons NW Nguyen C : Evidence that aging and amyloid promote microglial cell senescence. *Rejuvenation Res.* 2007;10(1):61–74. 10.1089/rej.2006.9096 17378753

[18] Saez-Atienzar S Masliah E : Author Correction: Cellular senescence and Alzheimer disease: the egg and the chicken scenario. *Nat Rev Neurosci.* 2020;21(10):587. 10.1038/s41583-020-0366-3 32792667

[19] Salminen A Ojala J Kaarniranta K : Astrocytes in the aging brain express characteristics of senescence-associated secretory phenotype. *Eur J Neurosci.* 2011;34(1):3–11. 10.1111/j.1460-9568.2011.07738.x 21649759

[20] Diniz BS Mulsant BH Reynolds CF 3rd : Association of Molecular Senescence Markers in Late-Life Depression With Clinical Characteristics and Treatment Outcome. *JAMA Netw Open.* 2022;5(6):e2219678. 10.1001/jamanetworkopen.2022.19678 35771573 PMC9247739

[21] Zhang L Pitcher LE Prahalad V : Targeting cellular senescence with senotherapeutics: senolytics and senomorphics. *FEBS J.* 2023;290(5):1362–1383. 10.1111/febs.16350 35015337

[22] Di Micco R Krizhanovsky V Baker D : Cellular senescence in ageing: from mechanisms to therapeutic opportunities. *Nat Rev Mol Cell Biol.* 2021;22(2):75–95. 10.1038/s41580-020-00314-w 33328614 PMC8344376

[23] Novais EJ Tran VA Johnston SN : Long-term treatment with senolytic drugs Dasatinib and Quercetin ameliorates age-dependent intervertebral disc degeneration in mice. *Nat Commun.* 2021;12(1):5213. 10.1038/s41467-021-25453-2 34480023 PMC8417260

[24] Alharbi KS Afzal O Altamimi ASA : A study of the molecular mechanism of quercetin and dasatinib combination as senolytic in alleviating age-related and kidney diseases. *J Food Biochem.* 2022;46(12): e14471. 10.1111/jfbc.14471 36268851

[25] Hickson LJ Langhi Prata LGP Bobart SA : Senolytics decrease senescent cells in humans: Preliminary report from a clinical trial of Dasatinib plus Quercetin in individuals with diabetic kidney disease. *EBioMedicine.* 2019;47:446–456. 10.1016/j.ebiom.2019.08.069 31542391 PMC6796530

[26] Justice JN Nambiar AM Tchkonia T : Senolytics in idiopathic pulmonary fibrosis: Results from a first-in-human, open-label, pilot study. *EBioMedicine.* 2019;40:554–563. 10.1016/j.ebiom.2018.12.052 30616998 PMC6412088

[27] Gonzales MM Garbarino VR Marques Zilli E : Senolytic Therapy to Modulate the Progression of Alzheimer's Disease (SToMP-AD): A Pilot Clinical Trial. *J Prev Alzheimers Dis.* 2022;9(1):22–29. 10.14283/jpad.2021.62 35098970 PMC8612719

[28] Nambiar A Kellogg D 3rd Justice J : Senolytics dasatinib and quercetin in idiopathic pulmonary fibrosis: results of a phase I, single-blind, single-center, randomized, placebo-controlled pilot trial on feasibility and tolerability. *EBioMedicine.* 2023;90: 104481. 10.1016/j.ebiom.2023.104481 36857968 PMC10006434

[29] Diniz BS Seitz-Holland J Sehgal R : Geroscience-Centric Perspective for Geriatric Psychiatry: Integrating Aging Biology With Geriatric Mental Health Research. *Am J Geriatr Psychiatry.* 2023;32:1–16. 10.1016/j.jagp.2023.09.014 37845116 PMC10841054

[30] Lorenzo EC Kuchel GA Kuo CL : Major depression and the biological hallmarks of aging. *Ageing Res Rev.* 2023;83: 101805. 10.1016/j.arr.2022.101805 36410621 PMC9772222

[31] Diniz BS Reynolds CF III Sibille E : Major depression and enhanced molecular senescence abnormalities in young and middle-aged adults. *Transl. Psychiatry.* 2019;9(1):198. 10.1038/s41398-019-0541-3 31434875 PMC6704136

[32] Diniz BS Reynolds CF III Sibille E : Enhanced Molecular Aging in Late-Life Depression: the Senescent-Associated Secretory Phenotype. *Am J Geriatr Psychiatry.* 2017;25(1):64–72. 10.1016/j.jagp.2016.08.018 27856124 PMC5164865

[33] Diniz BS Vieira EM Mendes-Silva AP : Mild cognitive impairment and major depressive disorder are associated with molecular senescence abnormalities in older adults. *Alzheimers Dement (N Y).* 2021;7(1): e12129. 10.1002/trc2.12129 33816758 PMC8012242

[34] Lorenzo EC Figueroa JE Demirci DA : Unraveling the association between major depressive disorder and senescent biomarkers in immune cells of older adults: a single-cell phenotypic analysis. *Front Aging.* 2024;5:1376086. 10.3389/fragi.2024.1376086 38665228 PMC11043554

[35] Diniz BS Lin CW Sibille E : Circulating biosignatures of late-life depression (LLD): Towards a comprehensive, data-driven approach to understanding LLD pathophysiology. *J Psychiatr Res.* 2016;82:1–7. 10.1016/j.jpsychires.2016.07.006 27447786 PMC9344393

[36] Domenici E Wille DR Tozzi F : Plasma protein biomarkers for depression and schizophrenia by multi analyte profiling of case-control collections. *PLoS One.* 2010;5(2):e9166. 10.1371/journal.pone.0009166 20161799 PMC2820097

[37] Rowland T Perry BI Upthegrove R : Neurotrophins, cytokines, oxidative stress mediators and mood state in bipolar disorder: systematic review and meta-analyses. *Br J Psychiatry.* 2018;213(3):514–525. 10.1192/bjp.2018.144 30113291 PMC6429261

[38] Diniz BS Mendes-Silva AP Silva LB : Oxidative stress markers imbalance in late-life depression. *J Psychiatr Res.* 2018;102:29–33. 10.1016/j.jpsychires.2018.02.023 29574402

[39] Vieira EL Mendes-Silva AP Ferreira JD : Oxidative DNA damage is increased in older adults with a major depressive episode: A preliminary study. *J Affect Disord.* Jan 15 2021;279:106–110. 10.1016/j.jad.2020.09.084 33045551

[40] Kirkland JL Tchkonia T : Senolytic drugs: from discovery to translation. *J Intern Med.* 2020;288(5):518–536. 10.1111/joim.13141 32686219 PMC7405395

[41] Riessland M Ximerakis M Jarjour AA : Therapeutic targeting of senescent cells in the CNS. *Nat Rev Drug Discov.* 2024;23(11):817–837. 10.1038/s41573-024-01033-z 39349637 PMC11927922

[42] Aguilar M Bhuket T Torres S : Prevalence of the metabolic syndrome in the United States, 2003-2012. *JAMA.* 2015;313(19):1973–1974. 10.1001/jama.2015.4260 25988468

[43] Mitchell AJ Vancampfort D Sweers K : Prevalence of Metabolic Syndrome and Metabolic Abnormalities in Schizophrenia and Related Disorders—A Systematic Review and Meta-Analysis. *Schizophrenia Bull.* 2011;39(2):306–318. 10.1093/schbul/sbr148 22207632 PMC3576174

[44] Forman DE Racette SB Toto PE : Modified Application of Cardiac Rehabilitation in Older Adults (MACRO) Trial: Protocol changes in a pragmatic multi-site randomized controlled trial in response to the COVID-19 pandemic. *Contemp Clin Trials.* 2022;112: 106633. 10.1016/j.cct.2021.106633 34823001 PMC8648552

[45] Konrad M Nieman DC . Evaluation of Quercetin as a Countermeasure to Exercise-Induced Physiological Stress. In: Lamprecht M , ed. *Antioxidants in Sport Nutrition.* 2015. 10.1201/b17442-10 26065092

[46] Pelletier DM Lacerte G Goulet ED : Effects of quercetin supplementation on endurance performance and maximal oxygen consumption: a meta-analysis. *Int J Sport Nutr Exerc Metab.* 2013;23(1):73–82. 10.1123/ijsnem.23.1.73 22805526

[47] Tkemaladze J , Apkhazava D: Dasatinib and Quercetin: Short-Term Simultaneous Administration Improves Physical Capacity In Human. *J Med Healthc.* 2019;1.

[48] Cortes JE Abruzzese E Chelysheva E : The impact of dasatinib on pregnancy outcomes. *Am J Hematol.* 2015;90(12):1111–1115. 10.1002/ajh.24186 26348106 PMC5115878

[49] Fossel M : Cell Senescence, Telomerase, and Senolytic Therapy. *OBM Geriatrics*. 2019;3(1):1. 10.21926/obm.geriatr.1901034 Reference Source

[50] Open Science Framework: Protocol for a pilot clinical trial of the senolytic drug combination Dasatinib plus Quercetin to mitigate age-related health and cognitive decline in mental disorders. 10.17605/OSF.IO/92GMQ PMC12120425 40443429

