# Supplementary material for: Protocol for a pilot clinical trial of the senolytic drug combination Dasatinib Plus Quercetin to mitigate age-related health and cognitive decline in mental disorders
Source: F1000Res. 2025 Mar 5;13:1072. Originally published 2024 Sep 19. [Version 2] doi: 10.12688/f1000research.151963.2 (PMC12120425; doi:10.12688/f1000research.151963.2)
Supplement: Peer review history

# Reviewer report: Reviewer response for version 2

**DOI:** 10.5256/f1000research.176084.r382393

**Published:** 2025-05-28

**Authors:** Wan-Ping Lee

**Affiliations:** 1 University of Pennsylvania, Philadelphia, PA, USA

The authors propose a pilot clinical trial investigating the safety, feasibility, and preliminary effects of the senolytic drug combination dasatinib plus quercetin (D+Q) in older adults with schizophrenia, schizoaffective disorder, or treatment-resistant depression (TRD). Thirty participants will receive intermittent D+Q dosing over four weeks, alongside lifestyle education. The study will assess changes in senescence-associated secretory phenotype (SASP) biomarkers, neuropsychological functioning, and brain imaging markers over a one-year period to explore whether D+Q can mitigate accelerated aging associated with serious mental illness.

 Major Comments:

 1. The statistical analysis plan is currently underdeveloped. While it is appropriate for a pilot study to emphasize feasibility, exploratory analyses still require transparency. I recommend the authors provide:

 - A rationale or justification for the chosen sample size of 30 participants;

 - A clear description of how effect sizes and confidence intervals will be calculated and interpreted;

 - A plan for handling missing data and participant dropouts;

 - Clarification on whether within-subject changes over time will be described visually or modeled analytically (e.g., through slopes or individual trajectories).

 Without these details, it is difficult to determine whether the study will generate results that meaningfully inform a larger, future trial.

 2. The decision to include only participants aged >50 years with schizophrenia/schizoaffective disorder may unnecessarily limit the eligible population. Although the rationale of accelerated biological aging is cited, schizophrenia often presents much earlier, and aging-related biomarkers such as elevated SASP may be present well before age 50. Broadening the age criteria could enhance both the feasibility of recruitment and the generalizability of findings.

 3. The inclusion of a lifestyle risk management component introduces a potential confounder, particularly given its likely influence on inflammation, cognition, and aging-related biomarkers. The absence of an analytic plan to monitor or adjust for this intervention complicates interpretation of D+Q’s effects. Greater clarity on how lifestyle adherence will be measured and accounted for is needed.

 4. The study involves multiple data modalities, including biological, neuroimaging, cognitive, and behavioral assessments, yet the protocol lacks a clear plan for integrating these findings. Even at an exploratory level, outlining how these data streams will be interpreted in relation to one another would strengthen the study's value and future utility.

 5. The protocol does not address biological sex or gender in recruitment, stratification, or analysis. Given well-established sex differences in both aging biology and psychiatric disorders, this omission could limit the interpretability and applicability of the findings. The authors are encouraged to explicitly incorporate sex/gender as a variable of interest.

Is the study design appropriate for the research question?

Yes

Is the rationale for, and objectives of, the study clearly described?

Yes

Are sufficient details of the methods provided to allow replication by others?

Partly

Are the datasets clearly presented in a useable and accessible format?

Yes

Reviewer Expertise:

genomic/genetic research for neurodegenetive diseases; bioinformatics

I confirm that I have read this submission and believe that I have an appropriate level of expertise to confirm that it is of an acceptable scientific standard, however I have significant reservations, as outlined above.

# Reviewer report: Reviewer response for version 2

**DOI:** 10.5256/f1000research.176084.r369668

**Published:** 2025-03-22

**Authors:** Amer Burhan

**Affiliations:** 1 Ontario Shores Centre for Mental Health Sciences, University of Toronto, Toronto, Ontario, Canada

The authors addressed my concerns adequately.

Is the study design appropriate for the research question?

Yes

Is the rationale for, and objectives of, the study clearly described?

Yes

Are sufficient details of the methods provided to allow replication by others?

Yes

Are the datasets clearly presented in a useable and accessible format?

Not applicable

Reviewer Expertise:

geriatric psychiatry, dementia, treatment resistant depression, brain stimulation and ketamine

I confirm that I have read this submission and believe that I have an appropriate level of expertise to confirm that it is of an acceptable scientific standard.

# Reviewer report: Reviewer response for version 1

**DOI:** 10.5256/f1000research.166660.r335596

**Published:** 2024-10-29

**Authors:** Pierfelice Cutrufelli

**Affiliations:** 1 University of Catania, Catania, Italy

This protocol describes an interesting pilot study investigating the potential of senolytic therapy in mental disorders associated with accelerated aging, using drug combination Dasatinib Plus Quercetin. While the overall design is thoughtful and the rationale well-presented, several methodological concerns and omissions need to be addressed.

 Literature Review

- The literature review regarding nutraceutical interventions in severe mental illness appears insufficient. While the authors focus on the senolytic properties of dasatinib plus quercetin (D+Q), they fail to adequately review the existing literature on quercetin and other nutraceuticals in psychiatric conditions. 
- The protocol's review of dasatinib is insufficient, lacking comprehensive discussion of its pharmacological properties as a tyrosine kinase inhibitor, its established safety profile in oncological and non-oncological conditions, and potential interactions with psychiatric medications and other commonly used drugs in the target population. Furthermore, while the authors cite its senolytic properties, they should provide a more thorough review of previous clinical trials using dasatinib as a senolytic agent.
- The protocol provides insufficient justification for the specific combination of dasatinib plus quercetin (D+Q), lacking detailed mechanistic explanation of how these compounds work individually (particularly dasatinib's role as a tyrosine kinase inhibitor and quercetin's anti-inflammatory and antioxidant properties) and the molecular basis for their proposed synergistic action in senolytic effects. The authors should thoroughly review both the pharmacological properties and existing clinical evidence for this combination, including potential interactions with psychiatric medications and the rationale for their specific dosing regimen, which is crucial for interpreting study outcomes and identifying relevant biomarkers. Additionally, the protocol should clearly explain why this particular combination might be especially beneficial in psychiatric populations, as opposed to other senolytic approaches.
- The protocol would benefit from a more comprehensive literature review and methodological considerations drawn from recent research in nutraceutical interventions for psychiatric and aging-related conditions. Several key studies have demonstrated the potential of natural compounds in these fields: Grosso et al. (2014) showed through meta-analysis that omega-3 supplementation significantly improves depressive symptoms, particularly as adjuvant therapy; Pivari et al. (2022) demonstrated how targeted nutraceutical interventions like curcumin can effectively modulate inflammation and oxidative stress pathways in chronic conditions; and Fusar-Poli et al. (2022) established that even simple interventions like cocoa-rich products can significantly impact depression and anxiety symptoms. Incorporating these and other relevant findings into your review could better consolidate your aims, making them plausible, relevant, and achievable.

SASP Definition and Measurement:

- The manuscript fails to clearly define the SASP (Senescence-Associated Secretory Phenotype) acronym at first use and does not adequately explain its biological significance
- The methodology for SASP measurement and evaluation, although mentioned, needs more details for clarity and transparency.
- The correlation between proposed outcome measures and SASP levels is not clearly explained

Assessment Procedures:

- The protocol lacks specificity regarding who will conduct the various assessments
- No information on rater training or inter-rater reliability measures
- Distinction between self-report and clinician-administered scales is not clear
- The rationale for selecting specific psychological/psychiatric measures is inadequately explained; a detailed description of the scales and their rationale is required

Confounding Factors:

- The concurrent lifestyle intervention could mask the effects of D+Q treatment. Address this, since no clear plan is explained for controlling or analyzing this potential confounding variable
- Exclusion criteria should address concurrent use of other nutraceuticals that might affect outcomes

Statistical Analysis:

- The statistical analysis plan is insufficiently detailed; while the authors state that "analysis for Aims 2 and 3 will be primarily descriptive in nature,. this appears insufficient as even pilot studies should include preliminary hypothesis testing to identify promising trends and guide future research. Moreover, the complex nature of longitudinal data with multiple outcomes and two distinct diagnostic groups (TRD vs. schizophrenia/schizoaffective disorder) demands more sophisticated statistical modeling, including mixed-effects models and interaction analyses, to properly inform future trial design.
- Effect size calculation is particularly crucial in biomedical research as it quantifies the magnitude of treatment impact, enables comparison across different interventions and studies through standardized measures, and provides essential information for determining sample sizes in future clinical trials - aspects that are especially relevant when testing novel compounds like senolytics where establishing clinical significance is as important as statistical significance.While the authors mention they will examine "effect size and 95% confidence interval for effects," they fail to specify which effect size measures will be used (e.g., Cohen's d, Hedges' g, or partial eta-squared for repeated measures), how these will be calculated for their longitudinal data structure, and what methods will be employed to handle the multiple comparison problem in effect size estimation across different outcomes.
- No clear strategy for handling missing data
- Lack of power calculations even for pilot study effect size estimation
- More detail is needed on your statistical approach to prevent reporting bias, such as multiple test corrections.

Assessment Tools:

- Better explanation needed for choice of specific scale. E.g. the authors will use the WHO Disability Assessment Schedule (WHODAS) but provide limited justification for this choice and lack detail in their analysis strategy. Recent research by Rodolico et al. (2024) demonstrated both the utility of WHODAS in evaluating disability in patients with severe mental illness and its significant correlation with medication side effects, providing a methodological framework that could inform this protocol. Given the potential interaction between existing antipsychotic medications and the proposed senolytic intervention, the authors could read and consider this recent evidence to strengthen their assessment approach and explain how WHODAS specifically relates to their intervention outcomes. This is particularly relevant as their protocol involves similar patient populations and could benefit from established methods for analyzing disability outcomes in the context of medication effects. A similar evaluation and reference standard should be conducted for each scale and measure, where necessary.
- Unclear how screening assessments will be integrated into analysis.
- Validation status of measures for the target populations not addressed.

Conclusion: While this protocol addresses an important research question with potential clinical significance, several methodological issues need to be addressed before implementation. The primary concerns relate to measurement protocols and scale measures description, potential confounding factors, and statistical analysis planning. A more comprehensive literature review is also needed. Addressing these issues would strengthen the study's scientific rigor and potential contribution to the field.

Is the study design appropriate for the research question?

Partly

Is the rationale for, and objectives of, the study clearly described?

Partly

Are sufficient details of the methods provided to allow replication by others?

Yes

Are the datasets clearly presented in a useable and accessible format?

Not applicable

Reviewer Expertise:

My primary area of expertise lies in clinical psychiatry, with a particular focus on schizophrenia and mood disorders. I specialize in evaluating the efficacy, safety, and pharmacological profiles of psychiatric drugs, including innovative pharmacological interventions. I also possess a strong background in conducting systematic reviews and meta-analyses, which allows me to critically assess evidence-based practices in psychiatric care.

I confirm that I have read this submission and believe that I have an appropriate level of expertise to confirm that it is of an acceptable scientific standard, however I have significant reservations, as outlined above.

## References

[1] : Omega-3 fatty acids and depression: scientific evidence and biological mechanisms. *Oxid Med Cell Longev* .2014;2014: 10.1155/2014/313570 313570 10.1155/2014/313570 24757497 PMC3976923

[2] : Curcumin Supplementation (Meriva®) Modulates Inflammation, Lipid Peroxidation and Gut Microbiota Composition in Chronic Kidney Disease. *Nutrients* .2022;14(1) : 10.3390/nu14010231 10.3390/nu14010231 35011106 PMC8747135

[3] : The effect of cocoa-rich products on depression, anxiety, and mood: A systematic review and meta-analysis. *Crit Rev Food Sci Nutr* .2022;62(28) : 10.1080/10408398.2021.1920570 7905-7916 10.1080/10408398.2021.1920570 33970709

[4] : Disability and Adverse Effects of Oral Versus Long-Acting Injectable Antipsychotics in Schizophrenia-Spectrum and Bipolar Disorder: A Comparison Based on Data-Driven Taxonomy. *Clin Drug Investig* .2024;44(9) : 10.1007/s40261-024-01391-x 715-727 10.1007/s40261-024-01391-x 39302586 PMC11455686

# Reviewer report: Reviewer response for version 1

**DOI:** 10.5256/f1000research.166660.r325468

**Published:** 2024-09-24

**Authors:** Amer Burhan

**Affiliations:** 1 Ontario Shores Centre for Mental Health Sciences, University of Toronto, Toronto, Ontario, Canada

This manuscript describes a protocol for a pilot study investigating the safety and preliminary efficacy of a senolytic intervention (D+Q), that has evidence in animal models and preliminary evidence of safety in human clinical conditions, in patients with schizophrenia/schizoaffective disorder and treatment resistant depression, at one center in the US.

 The rationale is well articulated and is sound building on this group's already established work on cell senescence proteomic biomarkers, that is done mainly in late life depression. The design is sound and will likely answer the safety question in this population and preliminary evidence of target engagement. The clinical variables and brain imaging are not likely to be adequately explored given the small sample and the mixed population to be recruited. It would have been more likely to achieve some of the third objective in terms of estimating effect size, if the authors focused on one of the populations at a time, in this case late life treatment resistant depression as better fit for with the previous results form this group. I suggest trying to recruit 30 participants with late life depression, which would make this more useful, that doesn't mean the group can't do preliminary assessment of the safety and target engagement in schizophrenia and schizoaffective disorder, but would allow better conclusions from each of the studies

 thanks you for inviting me to review this interesting protocol

Is the study design appropriate for the research question?

Yes

Is the rationale for, and objectives of, the study clearly described?

Yes

Are sufficient details of the methods provided to allow replication by others?

Yes

Are the datasets clearly presented in a useable and accessible format?

Not applicable

Reviewer Expertise:

geriatric psychiatry, dementia, treatment resistant depression, brain stimulation and ketamine

I confirm that I have read this submission and believe that I have an appropriate level of expertise to confirm that it is of an acceptable scientific standard, however I have significant reservations, as outlined above.
